# Development and validation of a clinical prediction model for first fall in early Parkinson’s disease: a study of two fall-naive cohorts

**DOI:** 10.3389/fnagi.2026.1735524

**Published:** 2026-02-17

**Authors:** Yu Wang, Jianing Mei, Yunzhe Tang, Hongping Zhao, Zijun Wei, Qingliang Tao, Xueyi Han, Jiyuan Hu, Yunyun Zhang

**Affiliations:** Department of Neurology, Yueyang Hospital of Integrated Traditional Chinese and Western Medicine, Shanghai University of Traditional Chinese Medicine, Shanghai, China

**Keywords:** early stage, falls, Parkinson’s disease, prognostic model, prospective studies

## Abstract

**Background:**

Falls are frequent and debilitating complications in Parkinson’s disease (PD), with a substantial risk present even in early stages. Predicting the first fall is critical for preventive interventions, yet existing models are often unsuitable for fall-naive, early PD patients due to their reliance on fall history.

**Objective:**

This study aimed to develop and externally validate a clinical prediction model for first falls in a population of fall-naive, early PD patients.

**Methods:**

This prognostic model study used data from two cohorts: the Parkinson’s Progression Markers Initiative (PPMI) for model development (*n* = 283) and internal validation (*n* = 120), and an independent Chinese cohort for external validation (*n* = 150). Participants were fall-naive with early PD (Hoehn and Yahr stage 1–2) and were followed for 36 months. The primary outcome was time to the first fall. A Cox proportional hazards model was developed using readily accessible clinical variables. Model performance was assessed using discrimination (C-index, AUC), calibration, and decision curve analysis.

**Results:**

During follow-up, 16.9% of PPMI participants and 20.7% of the Chinese cohort experienced a first fall. The final model incorporated five independent predictors: lower body mass index, asymptomatic orthostatic hypotension, lower Montreal Cognitive Assessment score, a Geriatric Depression Scale-15 score > 5, and a higher postural instability and gait disorder score. The model demonstrated good discrimination with an optimism-corrected C-index of 0.844 in the training set and maintained its performance in both internal (C-index: 0.768) and external validation (C-index: 0.825). Decision curve analysis indicated that the model demonstrated superior clinical net benefit for predicting falls over 36 months compared to 18 months.

**Conclusion:**

A history of falls is not necessary to predict the first fall in early PD. Our externally validated model, based on five easily ascertainable clinical factors, provides a practical tool for early risk stratification and can help guide individualized preventive strategies to delay or prevent initial falls in this vulnerable population.

## 1 Introduction

Parkinson’s disease (PD) is the second most common neurodegenerative disorder, with its prevalence increasing with global aging, thereby imposing a substantial disease burden (Ben-Shlomo et al., 2024). Falls are among the most frequent and disabling complications in individuals with PD, occurring at a much higher rate than in age-matched populations and often considered a key milestone in disease progression (Lindholm et al., 2021; Gonzalez et al., 2022; Camicioli et al., 2023). Falls can lead to severe physical injuries (e.g., fractures, traumatic brain injuries), psychological trauma (e.g., fear of falling, social isolation), functional decline (e.g., reduced quality of life, increased risk of institutionalization), and a significant healthcare-economic burden (Fasano et al., 2017; Paul et al., 2017).

While traditionally viewed as a predominant issue in mid-to-late-stage PD, accumulating evidence indicates a significantly elevated risk of falls even in early-stage PD (Bloem et al., 2001; Voss et al., 2012; Hiorth et al., 2017; Lord et al., 2017). Given that a first fall is the strongest predictor of subsequent recurrent falls (Pickering et al., 2007; van der Marck et al., 2014), identifying high-risk individuals before their initial fall and implementing preventive interventions is crucial. However, existing fall prediction models for PD have notable limitations that hinder their effective application in preventing first falls. Firstly, many models heavily rely on “fall history” as a core predictor (van der Marck et al., 2014; Lindholm et al., 2021; Murueta-Goyena et al., 2024), creating a logical paradox when predicting a first fall and rendering them inapplicable. Secondly, most studies focus on patients with mid-to-late-stage or mixed-duration PD, where key identified predictors [e.g., freezing of gait (FOG), dementia, orthostatic hypotension (OH)] have very low prevalence or are absent in early-stage patients (Lindholm et al., 2016; Malaguti et al., 2025). Thirdly, many existing studies employ cross-sectional or single-center cohort designs (Wood, 2002; Contreras and Grandas, 2012; Schrag et al., 2015), lacking large-scale prospective data and external validation, which limits model generalizability. Fourthly, some models depend on expensive or invasive biomarkers [e.g., positron emission tomography (PET) scans, cerebrospinal fluid (CSF) analysis, genotyping] (Isaias et al., 2020; Venuto et al., 2023), making them difficult to implement widely in routine clinical practice, particularly in many low- and middle-income countries (Li et al., 2019; Schiess et al., 2022).

Therefore, this study aimed to develop and internally validate a prediction model using data from the Parkinson’s Progression Markers Initiative (PPMI), a large, multicenter, prospective cohort, and to externally validate it using an independent local Chinese cohort. We focused specifically on fall-naive patients with early PD. The primary objective was to develop and validate a prognostic model based solely on readily available demographic and clinical variables to predict the risk of a first fall within 3 years in patients with early PD. This model is intended to empower clinicians to identify high-risk individuals at an early disease stage, enabling the implementation of targeted preventive strategies, thereby potentially delaying or preventing the first fall and improving long-term outcomes for patients with early PD.

## 2 Materials and methods

### 2.1 Study design and patient selection

This study involved the development and external validation of a clinical prognostic model. The reporting follows the Transparent Reporting of a multivariable prediction model for Individual Prognosis Or Diagnosis (TRIPOD) statement (Collins et al., 2015). Data for model development and internal validation were sourced from the prospective PPMI cohort. PPMI is a large, multicenter, longitudinal observational study initiated by The Michael J. Fox Foundation to identify PD progression biomarkers by enrolling drug-naive, early PD participants ^1^ (Parkinson Progression Marker Initiative, 2011). The PPMI study was approved by the institutional review board (IRB) at each participating site, and all participants provided written informed consent. Detailed inclusion and exclusion criteria for PPMI are described elsewhere (Parkinson Progression Marker Initiative, 2011). PPMI data (RRID:SCR_006431) were accessed by us in April 2025. For this study, we included de novo PD participants from the PPMI cohort who met the following criteria: (1) no history of falls at their baseline visit, based on fall questionnaire responses; (2) Hoehn and Yahr (H&Y) stage 1–2 at baseline; and (3) at least 36 months of follow-up data or follow-up until the occurrence of a first fall within this period. Participants with missing data for key variables or with comorbid conditions significantly affecting gait and balance (e.g., major stroke sequelae, peripheral neuropathy, myopathy, vertigo, visual impairment) were excluded from this analysis.

External validation data were sourced from an ongoing, single-center, prospective PD cohort initiated in 2022 at the Department of Neurology, Yueyang Hospital of Integrated Traditional Chinese and Western Medicine, Shanghai University of Traditional Chinese Medicine. Inclusion criteria were: (1) diagnosis of idiopathic PD according to the Movement Disorder Society (MDS) clinical diagnostic criteria (Postuma et al., 2015); (2) age 40–80 years; (3) H&Y stage 1–2 at enrollment; and (4) no reported PD-related falls before enrollment. Exclusion criteria were: (1) secondary parkinsonism (e.g., vascular, drug-induced, traumatic, normal pressure hydrocephalus, tumor-related) or parkinsonism-plus syndromes (e.g., progressive supranuclear palsy, multiple system atrophy); (2) comorbid conditions significantly affecting gait and balance; (3) prior deep brain stimulation therapy; and (4) severe dementia or psychiatric disorders impairing assessment or follow-up. This local study was approved by the Ethics Committee of Yueyang Hospital of Integrated Traditional Chinese and Western Medicine, Shanghai University of Traditional Chinese Medicine (IRB No. 2022-106), and all procedures were conducted in accordance with the principles of the Declaration of Helsinki. All participants provided written informed consent.

### 2.2 Clinical assessment

Baseline clinical data were collected at enrollment for both cohorts. For the PPMI cohort, demographic and medical history information was collected at baseline, including age, sex, years of education, body mass index (BMI), family history of parkinsonism, dominant symptom side, disease duration, and H&Y stage. (1) Motor symptom assessment: The severity of the four cardinal motor features of PD was assessed by summing relevant sub-items from the MDS-Unified Parkinson’s Disease Rating Scale (MDS-UPDRS) Parts II and III during the “off” medication state: bradykinesia score (items 3.2, 3.4–3.9, 3.14), tremor score (items 2.10, 3.15–3.18), rigidity score (item 3.3), and postural instability and gait disorder (PIGD) score (items 2.12–2.13, 3.10–3.12) (Goetz et al., 2008; Wolke et al., 2023). Patients were classified as tremor-dominant (TD) or non-TD based on the TD/PIGD mean ratio (Stebbins et al., 2013). (2) Non-motor symptom assessment: The 13 sub-items of MDS-UPDRS Part I were used to provide a general assessment of overall PD non-motor symptoms (Goetz et al., 2008). Given the very low scores for non-motor symptoms in early PD, which could lead to floor effects if raw scores were used directly, each sub-item was operationalized as a binary variable (score ≥ 1 indicating symptom presence, 0 indicating absence). The total score of the Scale for Outcomes in Parkinson’s disease for Autonomic Symptoms (SCOPA-AUT) was used to evaluate the level of autonomic dysfunction (Visser et al., 2004). Due to the skewed distribution of Geriatric Depression Scale-15 (GDS-15) scores in early PD, a score > 5 was considered indicative of at least mild depressive mood (Shin et al., 2019). Anxiety levels were assessed using the State-Trait Anxiety Inventory (STAI) and its subscales (Oei et al., 1990). Global cognitive function was evaluated using the adjusted Montreal Cognitive Assessment (MoCA) score (Nasreddine et al., 2005; Lu et al., 2011). The presence of hyposmia was assessed using the revised University of Pennsylvania Smell Identification Test (UPSIT), with a UPSIT percentile ≤ 15% considered indicative of hyposmia (Brumm et al., 2023). Sleep-wake dysfunction was assessed using the Epworth Sleepiness Scale (ESS) and the REM Sleep Behavior Disorder Screening Questionnaire (RBDSQ) (Kumar, 2003; Stiasny-Kolster et al., 2007). Asymptomatic orthostatic hypotension (OH) was defined as a drop in systolic blood pressure of ≥ 20 mmHg or diastolic blood pressure of ≥ 10 mmHg within 1–3 min of standing relative to supine values, provided that the MDS-UPDRS Part I Item 1.12 (“Lightheadedness on Standing”) score was 0. This combination confirmed the presence of orthostatic hypotension in the absence of typical symptoms (Beach and McKay, 2024). Further details on these variables have been described in previous publications (Parkinson Progression Marker Initiative, 2011).

To ensure comparability between cohorts, the local cohort followed operational procedures consistent with PPMI for the collection of demographic information and primary clinical data. However, a few differences existed. Firstly, for olfactory function assessment, the local cohort used subjective reporting rather than the objective UPSIT used in PPMI. Secondly, unlike the PPMI cohort where participants were drug-naive at baseline, most patients in the local cohort had initiated levodopa replacement therapy at enrollment; therefore, their baseline levodopa equivalent daily dose (LEDD) was recorded, calculated according to established methods (Tomlinson et al., 2010; Jost et al., 2023). All motor assessments in both cohorts were conducted during a practically defined “off” medication state to minimize the influence of anti-PD medications on assessment results.

### 2.3 Survival outcome

The primary outcome of this study was the time (in months) from baseline visit to the first fall experienced by PD patients within a 36-month follow-up period. A fall was defined according to widely accepted criteria: an unexpected event in which the participant comes to rest on the ground, floor, or other lower level (van der Marck et al., 2014; Montero-Odasso et al., 2022). This study focused on the first fall event, excluding near falls and not differentiating subsequent recurrent falls. In the PPMI cohort, fall information was collected using a standardized questionnaire at each regular follow-up visit (typically every 6 months) (Parkinson Progression Marker Initiative, 2011). In the local cohort, fall data were collected prospectively through structured interviews during routine clinical follow-ups (typically every 3 months). The time origin for the study was the date of the baseline visit for the PPMI cohort and the date of the first completed assessment for the local cohort. All participants included in the final analysis were successfully followed for 36 months or until their first fall, with no deaths or loss to follow-up for other reasons. Therefore, standard survival analysis methods were employed, and specific models for competing risks were not required. For participants who did not experience a first fall during the follow-up period, their data were right-censored at 36 months, and their follow-up time was recorded as 36 months.

### 2.4 Statistical analysis

To address potential bias from missing data in the PPMI cohort, multiple imputation was performed using the “mice” R package (version 3.17.0). Approximately 86% of participants had complete data for all candidate predictors. For variables with missing values, logistic regression imputation (“logreg”) was used for dominant side (missingness ∼12%) and UPSIT (∼3%), while predictive mean matching (“pmm”) was used for disease duration (∼11%). Five complete imputed datasets (*m* = 5) were generated with 10 iterations each. Iteration trace plots and density plots of imputed data showed good convergence and plausible distributions of imputed values, indicating satisfactory imputation quality (Supplementary Figures 1A–C). Subsequent statistical analyses were performed on these five imputed datasets, and results were pooled using Rubin’s rules.

Continuous variables were described as mean ± standard deviation (SD) or median [interquartile range (IQR)] based on their distribution, while categorical variables were presented as counts (n) and percentages (%). Between-group comparisons were performed using independent samples *t*-tests, Mann-Whitney U tests, chi-squared tests, or Fisher’s exact tests, as appropriate. Each of the five imputed PPMI datasets was randomly split into a training set (70%) and an internal validation set (30%). In each imputed training set, univariable Cox proportional hazards regression analysis was performed for all candidate predictors. Variables with a *p* < 0.05 in at least three of the five imputed datasets were included in the candidate pool for the multivariable model. These selected variables were then entered into a multivariable Cox proportional hazards regression model. A backward stepwise selection strategy based on the Akaike Information Criterion (AIC) was used to identify the most parsimonious and statistically significant combination of independent predictors. The Bayesian Information Criterion (BIC) was also calculated to assist in model comparison. Hazard ratios (HRs) and their 95% confidence intervals (CIs) were reported for each factor. A nomogram was constructed based on the final multivariable Cox model to serve as a visual tool for clinicians to estimate the 36-month probability of a first fall in fall-naive, early PD patients. The model’s overall performance was assessed in the training set, internal validation set, and external validation cohort based on discrimination, calibration, and clinical utility at 18 and 36 months. Overall discrimination was evaluated using Harrell’s C-index. Time-dependent C-indices and time-dependent areas under the receiver operating characteristic curve (AUCs) were calculated at 18 and 36 months to assess predictive accuracy at specific time points. An optimism-corrected C-index was calculated for internal validation via bootstrapping (500 resamples). Calibration was assessed by generating calibration curves at 18 and 36 months. Decision curve analysis (DCA) was used to evaluate the net benefit of the model across a range of threshold probabilities at 18 and 36 months. To further assess the model’s risk stratification capacity, patients were categorized into high-risk and low-risk groups based on the median of their predicted risk scores. Kaplan-Meier survival curves for time to first fall were plotted and compared between these groups using the log-rank test. All statistical analyses were performed using R software (version 4.5.0; The R Foundation for Statistical Computing, Vienna, Austria)^2^ and relevant packages. A two-sided *p* < 0.05 was considered statistically significant.

## 3 Results

### 3.1 Baseline characteristics of participants

**FIGURE 1:**
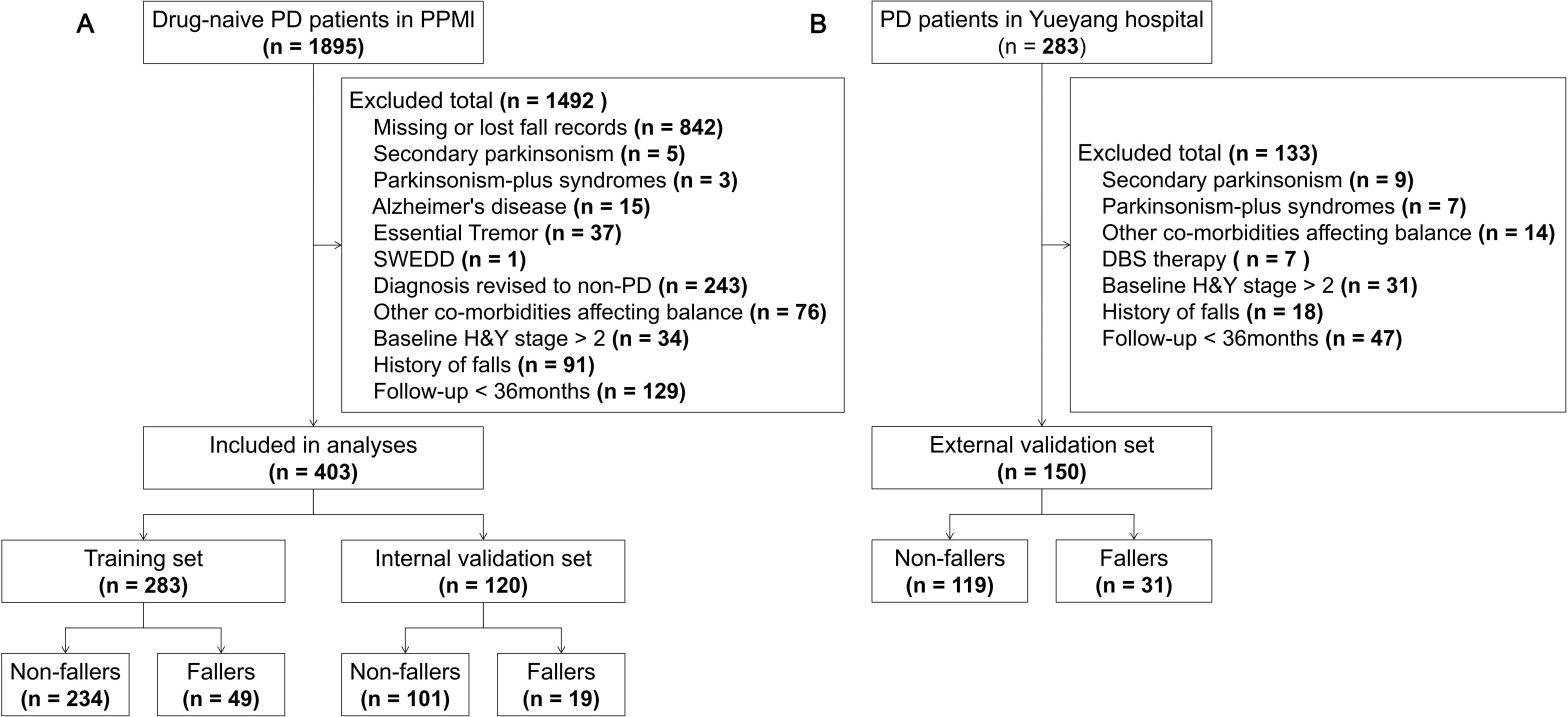
Flowchart of participant selection.

A total of 403 patients from the PPMI cohort and 150 patients from the local cohort were included in this study; the screening process is detailed in Figure 1. Patients in the PPMI cohort were randomly divided into a training set (*n* = 283) and an internal validation set (*n* = 120). No statistically significant differences were observed between these two sets for any baseline characteristics (all *P* > 0.05) (Supplementary Table 1). Significant differences in multiple baseline characteristics were observed between the total PPMI cohort and the local external validation cohort (Table 1). Compared to the PPMI cohort, PD patients in the local cohort were older, had fewer years of education, lower BMI, and a lower proportion of males. Regarding clinical features, patients in the local cohort had a longer disease duration, a higher proportion of H&Y stage 2 and non-tremor-dominant phenotype, and a lower proportion with a family history of parkinsonism. Motor function assessments revealed that patients in the local cohort had higher MDS-UPDRS Part II and Part III scores. For non-motor symptoms, patients in the local cohort had higher MDS-UPDRS Part I scores and SCOPA-AUT scores, but lower MoCA scores. Concurrently, the prevalence of GDS score > 5 and asymptomatic OH was also higher in the local cohort.

**TABLE 1:** Demographic and clinical characteristics of PPMI and Local cohort patients at baseline.

Variables	Overall (*n* = 553)	PPMI cohort (*n* = 403)	Local cohort (*n* = 150)	*P*-value
**Demographics**				
Age, years	65.00 (57.85, 70.00)	62.75 (55.89, 68.70)	69.00 (66.00, 73.75)	< 0.001
Sex, male, n(%)	339 (61.30%)	263 (65.26%)	76 (50.67%)	**0.002**
Education, years	16.00 (12.00, 18.00)	16.00 (14.00, 18.00)	12.00 (9.00, 15.00)	**< 0.001**
BMI, kg/m^2^	25.34 (23.44, 28.04)	25.86 (24.09, 28.92)	23.97 (22.24, 26.53)	**< 0.001**
**Clinical characteristics**				
Disease duration, years	1.32 (0.50, 3.00)	0.81 (0.38, 1.90)	3.00 (2.00, 4.38)	**< 0.001**
H&Y stage, n(%)				**0.003**
Stage 1	216 (39.06%)	173 (42.93%)	43 (28.67%)	
Stage 2	337 (60.94%)	230 (57.07%)	107 (71.33%)	
Family history, n (%)	180 (32.55%)	143 (35.48%)	37 (24.67%)	**0.021**
Dominant side, right, n(%)	293 (52.98%)	215 (53.35%)	78 (52.00%)	0.852
Motor subtype, n(%)				**0.012**
TD	344 (62.21%)	264 (65.51%)	80 (53.33%)	
Non-TD	209 (37.79%)	139 (34.49%)	70 (46.67%)	
MDS-UPDRS Part I	6.00 (3.00, 9.00)	5.00 (3.00, 7.00)	9.00 (7.00, 12.00)	**< 0.001**
Cognitive impairment, n(%)	149 (26.94%)	86 (21.34%)	63 (42.00%)	**< 0.001**
Hallucinations and psychosis, n(%)	31 (5.61%)	11 (2.73%)	20 (13.33%)	**< 0.001**
Depressed mood, n(%)	139 (25.14%)	93 (23.08%)	46 (30.67%)	0.086
Anxious mood, n(%)	212 (38.34%)	72 (48.00%)	140 (34.74%)	**0.006**
Apathy, n(%)	168 (30.38%)	63 (15.63%)	105 (70.00%)	**< 0.001**
DDS, n(%)	96 (17.36%)	84 (20.84%)	12 (8.00%)	**0.001**
Sleep problems, n(%)	357 (64.56%)	234 (58.06%)	123 (82.00%)	**< 0.001**
Daytime sleepiness, n(%)	335 (60.58%)	116 (77.33%)	219 (54.34%)	**< 0.001**
Pain, n(%)	301 (54.43%)	225 (55.83%)	76 (50.67%)	0.323
Urinary problems, n(%)	262 (47.38%)	184 (45.66%)	78 (52.00%)	0.218
Constipation problems, n(%)	251 (45.39%)	134 (33.25%)	117 (78.00%)	**< 0.001**
Light headedness, n(%)	136 (24.59%)	104 (25.81%)	32 (21.33%)	0.330
Fatigue, n(%)	280 (50.63%)	192 (47.64%)	88 (58.67%)	**0.027**
MDS-UPDRS Part II	6.00 (3.00, 9.00)	4.00 (2.00, 7.00)	9.00 (7.00, 12.00)	**< 0.001**
MDS-UPDRS Part III	20.00 (14.00, 27.00)	18.00 (12.00, 25.50)	24.00 (19.00, 28.00)	**< 0.001**
Tremor score	4.00 (2.00, 7.00)	5.00 (2.00, 7.00)	4.00 (2.00, 6.00)	0.308
PIGD score	1.00 (0.00, 3.00)	1.00 (0.00, 2.00)	5.00 (3.00, 7.00)	**< 0.001**
Rigidity score	3.00 (1.00, 6.00)	3.00 (1.00, 5.00)	5.00 (3.00, 7.00)	**< 0.001**
Bradykinsia score	8.00 (5.00, 11.00)	8.00 (5.00, 13.00)	7.00 (4.00, 10.00)	**< 0.001**
SCOPA-AUT	10.00 (7.00, 15.00)	9.00 (5.50, 12.50)	15.00 (11.00, 17.00)	**< 0.001**
GDS > 5, n(%)	85 (15.37%)	54 (13.40%)	31 (20.67%)	**0.048**
STAI	61.00 (50.00, 73.00)	59.00 (48.00, 74.00)	64.00 (58.00, 71.00)	**0.002**
STAI state	30.00 (24.00, 37.00)	29.00 (23.00, 38.00)	31.00 (28.00, 34.00)	**0.038**
STAI trait	31.00 (25.00, 37.00)	30.00 (24.00, 37.00)	33.00 (30.00, 37.00)	**< 0.001**
LEDD, mg	375.00 (300.00, 487.38)	NA	375.00 (300.00, 487.38)	NA
**Clinical characteristics**				
MoCA	27.00 (25.00, 28.00)	27.00 (26.00, 29.00)	24.00 (22.00, 27.00)	< 0.001
ESS	5.00 (3.00, 7.00)	5.00 (3.00, 7.00)	5.00 (2.00, 7.00)	0.164
RBDSQ	3.00 (2.00, 5.00)	3.00 (2.00, 5.00)	3.00 (2.00, 5.00)	0.661
Hyposmia, n(%)	380 (68.72%)	275 (68.24%)	105 (70.00%)	0.769
Asymptomatic OH, n(%)	100 (18.08%)	58 (14.39%)	42 (28.00%)	**< 0.001**
LEDD, mg	375.00 (300.00, 487.38)	NA	375.00 (300.00, 487.38)	NA

Data are presented as median (Q1, Q3), or number (%). PPMI, the Parkinson’s Progression Markers Initiative; BMI, body mass index, H&Y stage, Hoehn and Yahr stage; TD, tremor dominant; MDS-UPDRS, the MDS-sponsored revision of the unified Parkinson’s disease rating scale; DDS, dopamine dysregulation syndrome; PIGD, postural instability and gait difficulty; SCOPA-AUT, scales for outcomes in Parkinson’s disease-autonomic dysfunction; GDS, geriatric depression scale; STAI, State-Trait anxiety index; MoCA, Montreal cognitive assessment; ESS, Epworth sleepiness scale; RBDSQ, REM sleep behavior disorder screening questionnaire; OH, orthostatic hypotension; LEDD, levodopa equivalent daily dose. Bold values indicate statistical significance (*P* < 0.05).

### 3.2 Incidence of first falls during follow-up

**FIGURE 2:**
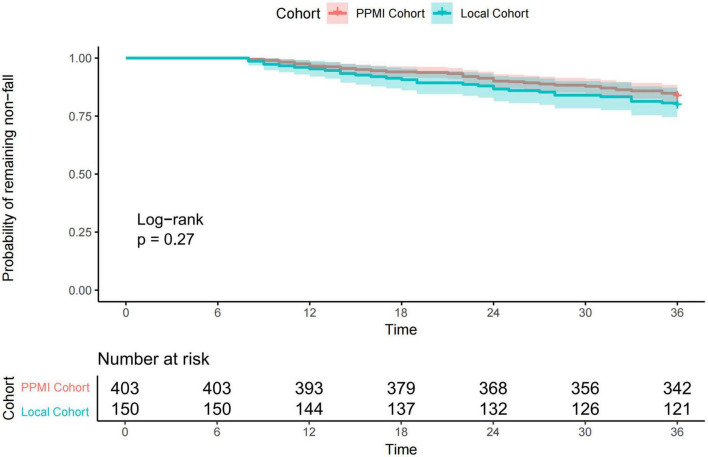
Kaplan-Meier curves for time to first fall in the PPMI cohort and the Local cohort.

During the 36-month follow-up period, 68 (16.9%) patients in the PPMI cohort experienced a first fall. The Kaplan-Meier estimated cumulative incidence of first falls within 3 years in the PPMI cohort was 16.9% (95% CI: 13.1–20.5%), with a median time to first fall of 23.0 months (IQR: 14.0–31.0 months). Similarly, within the 36-month follow-up period, 31 (20.7%) patients in the local cohort experienced a first fall. Their 3-year cumulative incidence of first falls was 20.7% (95% CI: 13.9–26.9%), with a median time to first fall of 19.0 months (IQR: 13.5–28.0 months). Although the 3-year cumulative fall incidence was slightly higher in the local cohort than in the PPMI cohort, this difference did not reach statistical significance (log-rank *P* = 0.27) (Figure 2).

### 3.3 Variable selection and model development

**FIGURE 3:**
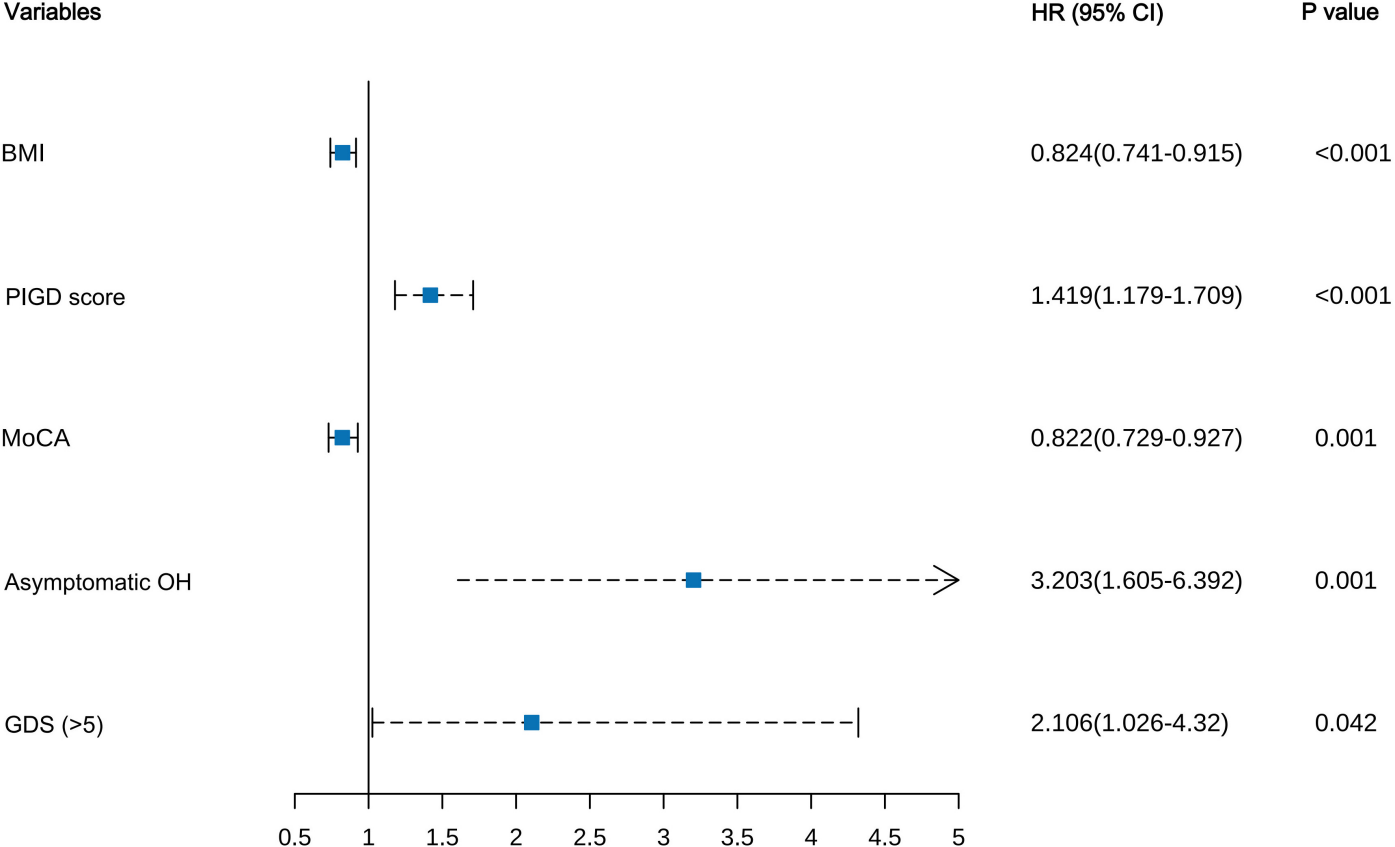
Forest plot of the final multivariable Cox regression model.

**FIGURE 4:**
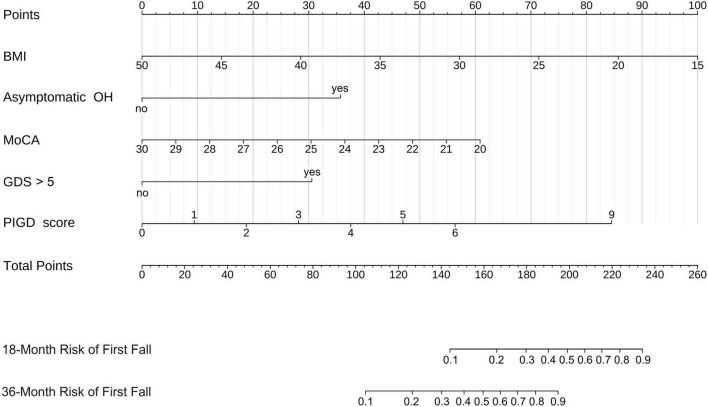
Nomogram for predicting 18 and 36-month risk of first fall.

In the PPMI training set, univariable Cox proportional hazards regression analysis was initially performed on all candidate demographic and clinical variables to screen for potential predictors (Supplementary Table 2). A total of 13 variables were significantly associated with the risk of a first fall in the univariable Cox analysis and were subsequently considered for the multivariable Cox proportional hazards regression model. The 13 variables found to be significant in univariable Cox analysis were subsequently entered into a multivariable Cox proportional hazards regression model. After initial fitting and assessment of this multivariable model, 7 candidate predictors were retained for further selection procedures (Table 2). “Disease duration” did not reach the predefined statistical significance level (*P* = 0.164) and was thus excluded first. Among the remaining 6 statistically significant predictors, “Hallucinations and psychosis” although statistically associated with fall risk, had an extremely low prevalence in early PD patients, accounting for only 3.18% in our training cohort. We compared the overall performance of Model 1 (including this variable) and Model 2 (excluding this variable), with results presented in Supplementary Table 3. Model 1 showed slightly better AIC, Bayesian Information Criterion (BIC), and C-index in the training set, but Model 2 had a slightly higher C-index in the internal validation set (0.768 vs. 0.763), suggesting that Model 1 might be slightly overfitted. Due to the limited improvement in predictive performance and its potential to diminish model robustness, “Hallucinations and psychosis,” was removed from the model. Ultimately, our clinical prediction model incorporated 5 independent predictors: lower BMI, presence of asymptomatic OH, lower MoCA score, GDS score > 5, and higher PIGD score (Figure 3). The variance inflation factor for all variables in the final model was less than 5, indicating no multicollinearity. Based on the Schoenfeld residuals test, all covariates in the model met the proportional hazards assumption (all *P* > 0.05), and the global test for the proportional hazards assumption yielded *P* = 0.068. For ease of clinical application, the final model was visualized as a nomogram (Figure 4). By locating the corresponding values on their respective axes to obtain points, summing these points to get a total score, which allows for the estimation of an individual’s 18 and 36-month risk of a first fall from the corresponding risk prediction axes.

**TABLE 2:** Univariable and multivariable Cox regression analyses in the PPMI training cohort.

Variables	Univariate cox regression		Multivariate cox regression			
	HR (95% CI)	*P*-value	Model 1^a^		Model 2^b^	
			HR (95% CI)	*P*-value	HR (95% CI)	*P*-value
BMI	0.789 (0.709–0.878)	**< 0.001**	0.819 (0.729–0.92)	**0.001**	0.824 (0.741–0.915)	**< 0.001**
Disease duration	0.504 (0.351–0.724)	**< 0.001**	0.728 (0.465–1.139)	0.164	–	–
Motor subtype (Non-TD)	2.486 (1.416–4.365)	**0.002**	–	–	–	–
Hallucinations and psychosis	4.158 (1.647–10.498)	**0.003**	4.644 (1.617–13.332)	**0.004**	–	–
Constipation problems	2.095 (1.196–3.668)	**0.010**	–	–	–	–
PIGD score	1.655 (1.437–1.906)	**< 0.001**	1.299 (1.02–1.655)	**0.034**	1.419 (1.179–1.709)	**< 0.001**
Rigidity score	1.136 (1.026–1.258)	**0.014**	–	–	–	–
SCOPA AUT	1.058 (1.02–1.097)	**0.003**	–	–	–	–
GDS ( > 5)	4.773 (2.645–8.612)	**< 0.001**	2.245 (1.066–4.728)	**0.033**	2.106 (1.026–4.32)	**0.042**
STAI trait	1.03 (1.002–1.058)	**0.037**	–	–	–	–
MoCA	0.732 (0.658–0.815)	**< 0.001**	0.809 (0.715–0.916)	**0.001**	0.822 (0.729–0.927)	**0.001**
RBDSQ	1.113 (1.028–1.204)	**0.008**	–	–	–	–
Asymptomatic OH	7.769 (4.425–13.64)	**< 0.001**	3.447 (1.738–6.835)	**< 0.001**	3.203 (1.605–6.392)	**0.001**

HR, hazard ratio; CI, confidence interval (95%); BMI, body mass index, TD, tremor dominant; PIGD, postural instability and gait difficulty; SCOPA-AUT, scales for outcomes in Parkinson’s disease-autonomic dysfunction; GDS, geriatric depression scale; STAI, State-Trait anxiety index; MoCA, Montreal cognitive assessment; RBDSQ, REM sleep behavior disorder screening questionnaire; OH, orthostatic hypotension.

*^a^*The Model 1 included BMI, PIGD, MoCA, GDS, asymptomatic OH, hallucinations and psychosis.

*^b^*The Model 2 included BMI, PIGD, MoCA, GDS, asymptomatic OH. Bold values indicate statistical significance (*P* < 0.05).

### 3.4 Model performance assessment and validation

**FIGURE 5:**
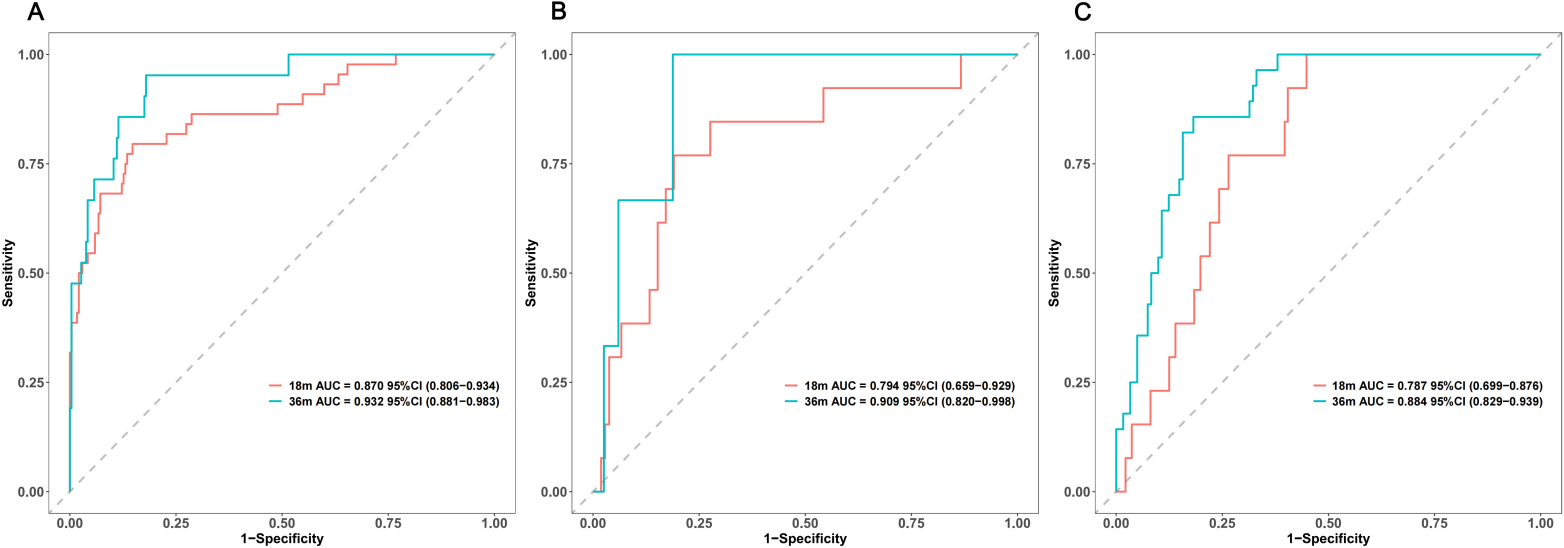
Time-dependent ROC curves for the prediction model at 18 and 36 months in the **(A)** PPMI training set, **(B)** PPMI internal validation set, and **(C)** local external validation cohort.

In the PPMI training set, the C-index for the prediction model was 0.847. Internal validation using 500 bootstrap resamples estimated an optimism of 0.003, resulting in an optimism-corrected C-index of 0.844 (95% CI: 0.786–0.907). In the PPMI internal validation set, the model’s C-index was 0.768 (95% CI: 0.656–0.880). In the local external validation cohort, the C-index was 0.825 (95% CI: 0.768–0.882). Overall and time-specific discriminative performance metrics, including C-indices and AUCs for all datasets, are summarized in Table 3. To further evaluate the model’s discrimination at specific time points, time-dependent ROC curves were plotted. At 18 months, the AUCs for the model in the PPMI training set (Figure 5A), internal validation set (Figure 5B), and external validation set (Figure 5C) were 0.870 (95% CI: 0.806–0.934), 0.794 (95% CI: 0.659–0.929), and 0.787 (95% CI: 0.699–0.876), respectively. At 36 months, the corresponding AUCs were 0.932 (95% CI: 0.881–0.983), 0.909 (95% CI: 0.820–0.998), and 0.884 (95% CI: 0.829–0.939) in the PPMI training set, internal validation set, and external validation set, respectively. These results indicate that the model demonstrated good to excellent discrimination across the training, internal validation, and external validation cohorts, particularly for predicting longer-term first fall risk.

**TABLE 3:** Discriminative performance of the prediction model in different datasets.

Performance metric	Time point	Training Set (*n* = 283)	Internal validation set (*n* = 120)	Local cohort (*n* = 150)
C-index (95% CI)	Overall	0.844 (0.786–0.907)	0.768 (0.656–0.880)	0.825 (0.768–0.882)
Time-dependent AUC (95% CI)	18-month	0.870 (0.806–0.934	0.794 (0.659–0.929)	0.787 (0.699–0.876)
Time-dependent AUC (95% CI)	36-month	0.932 (0.881–0.983)	0.909 (0.820–0.998)	0.884 (0.829–0.939)

C-index, concordance index; CI, confidence interval; AUC, Area under the curve.

**FIGURE 6:**
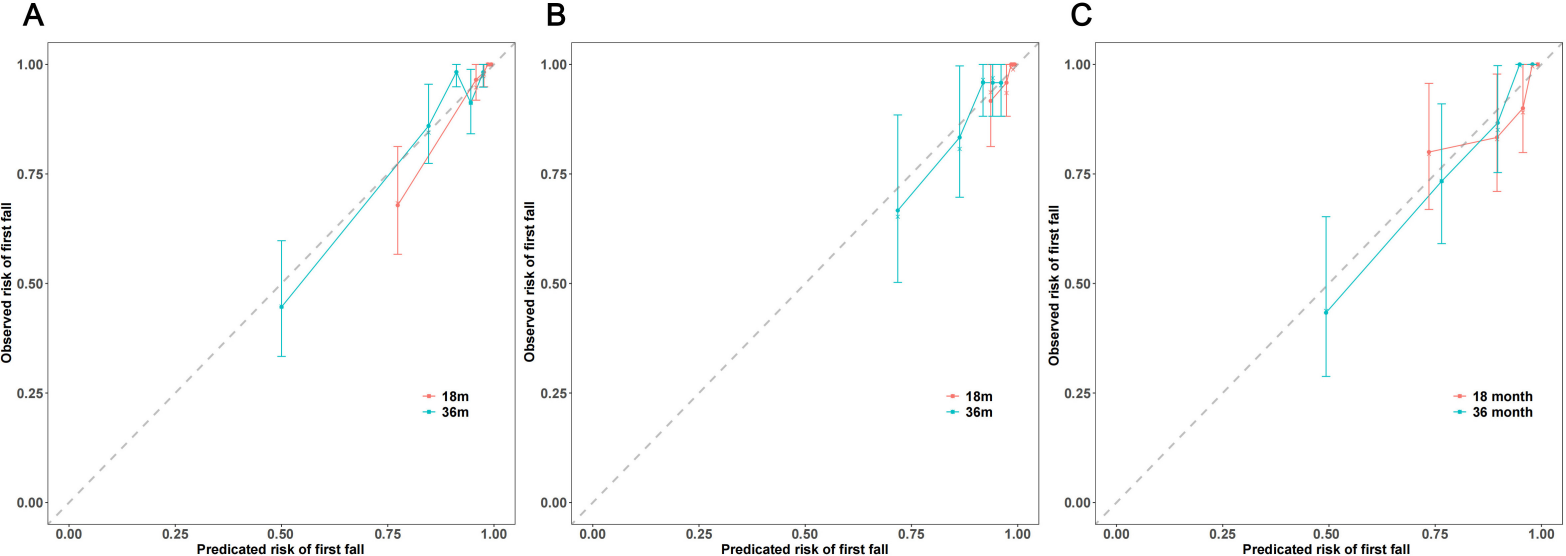
Calibration plots for the prediction model at 18 and 36 months in the **(A)** PPMI training set, **(B)** PPMI internal validation set, and **(C)** local external validation cohort.

Calibration curves for predicting 18 and 36-month first fall risk were generated for the training set, internal validation set, and external validation cohort, with confidence intervals estimated using 500 bootstrap resamples. For 18-month first fall risk prediction, the calibration curve for the PPMI training set (Figure 6A) showed good agreement across most risk strata, with slight deviation only at very high predicted risks ( > 0.75). The internal validation set (Figure 6B) showed acceptable calibration in the high-risk range. The 18-month calibration curve for the external validation cohort (Figure 6C) exhibited some fluctuation at predicted risks above 0.7. For 36-month first fall risk prediction, the calibration curve in the PPMI training set (Figure 6A) closely followed the ideal diagonal, demonstrating excellent calibration. In the PPMI internal validation set (Figure 6B) and the local external validation cohort (Figure 6C), the calibration curves also showed good agreement with the ideal line in the medium-to-high risk strata, although observed fall risk was slightly lower than predicted in the moderate-risk range. Overall, the calibration curves suggest that the model achieved acceptable calibration for predicting 36-month first fall risk across the three datasets, as predicted probabilities generally aligned with observed fall frequencies. However, for 18-month interim risk prediction, the model’s calibration was less consistent across datasets, with some deviation between predicted and observed values in certain risk strata, particularly in the external validation cohort.

**FIGURE 7:**
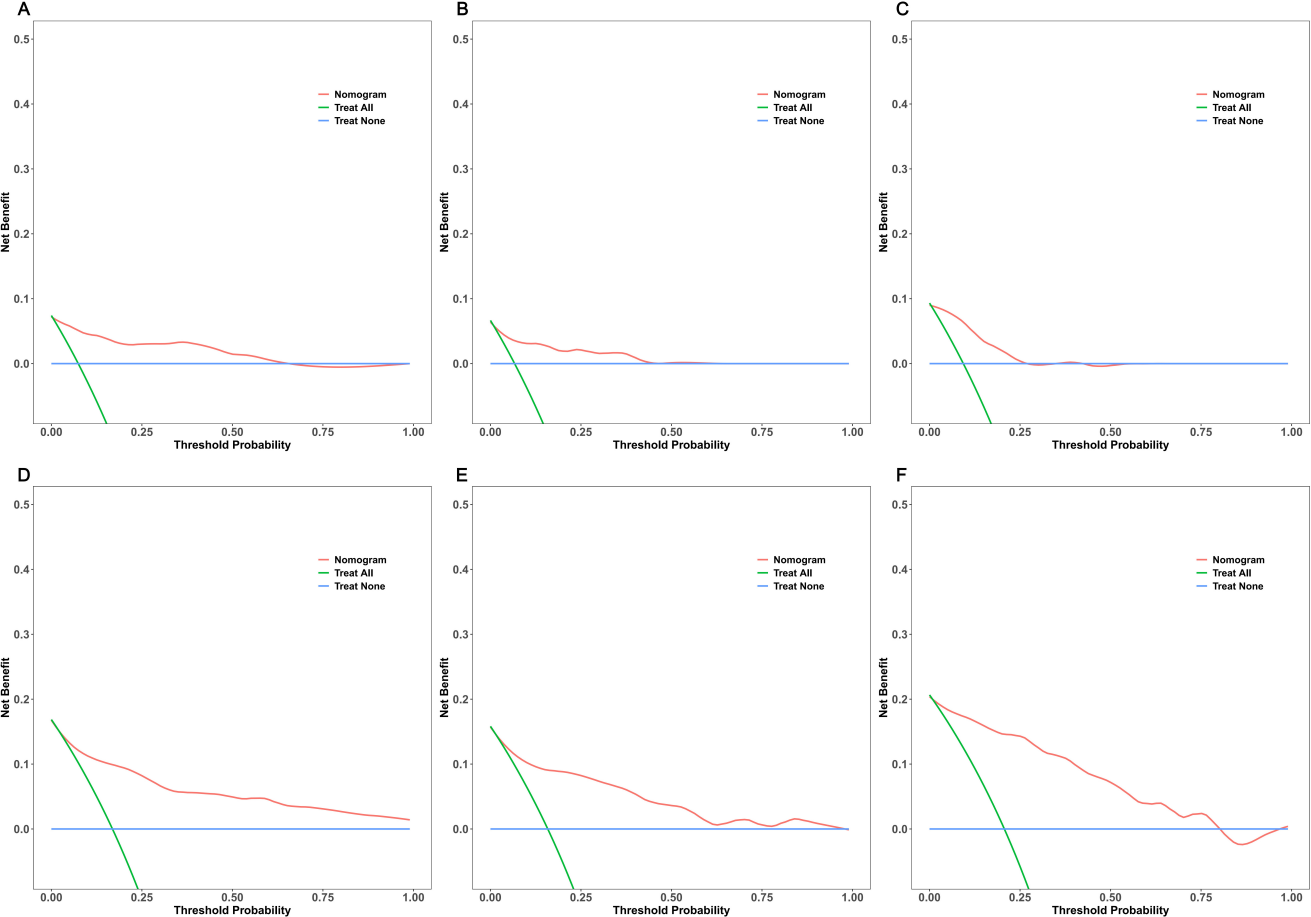
Decision curve analysis (DCA) for the prediction model. 18-month risk prediction in the **(A)** PPMI training set, **(B)** PPMI internal validation set, **(C)** local external validation cohort, 36-month risk prediction in the **(D)** PPMI training set, **(E)** PPMI internal validation set, and **(F)** local external validation cohort.

DCA was employed to evaluate the net benefit of using the model to guide clinical decisions across different threshold probabilities. For 18-month first fall risk prediction, the model demonstrated some clinical net benefit in all three datasets. In the PPMI training set (Figure 7A), internal validation set (Figure 7B), and external validation cohort (Figure 7C), using the model was superior to the “treat all” or “treat none” strategies within threshold probability ranges of approximately 0.05–0.65, 0.05–0.45, and 0.05–0.35, respectively, with corresponding maximum net benefits of approximately 0.07, 0.08, and 0.06. Compared to 18-month predictions, the model showed superior and more robust clinical utility for predicting 36-month first fall risk. In the PPMI training set (Figure 7D), the model provided a net benefit over the two extreme strategies across a very wide range of threshold probabilities (0.05–0.95), with a maximum net benefit of about 0.16. In the PPMI internal validation set (Figure 7E), the model was beneficial within a threshold range of approximately 0.05–0.74, achieving a maximum net benefit of about 0.18. In the local external validation cohort (Figure 7F), the model demonstrated clinical net benefit within a threshold range of approximately 0.05–0.75, also with a maximum value of about 0.18. Overall, the DCA results indicate that this prediction model, particularly for 36-month first fall risk, provides a net benefit across a wide range of clinical decision thresholds in all three datasets, suggesting good clinical utility. However, for 18-month interim predictions, the range and magnitude of net benefit provided by the model were relatively limited.

### 3.5 Assessment of model’s risk stratification capacity

**FIGURE 8:**
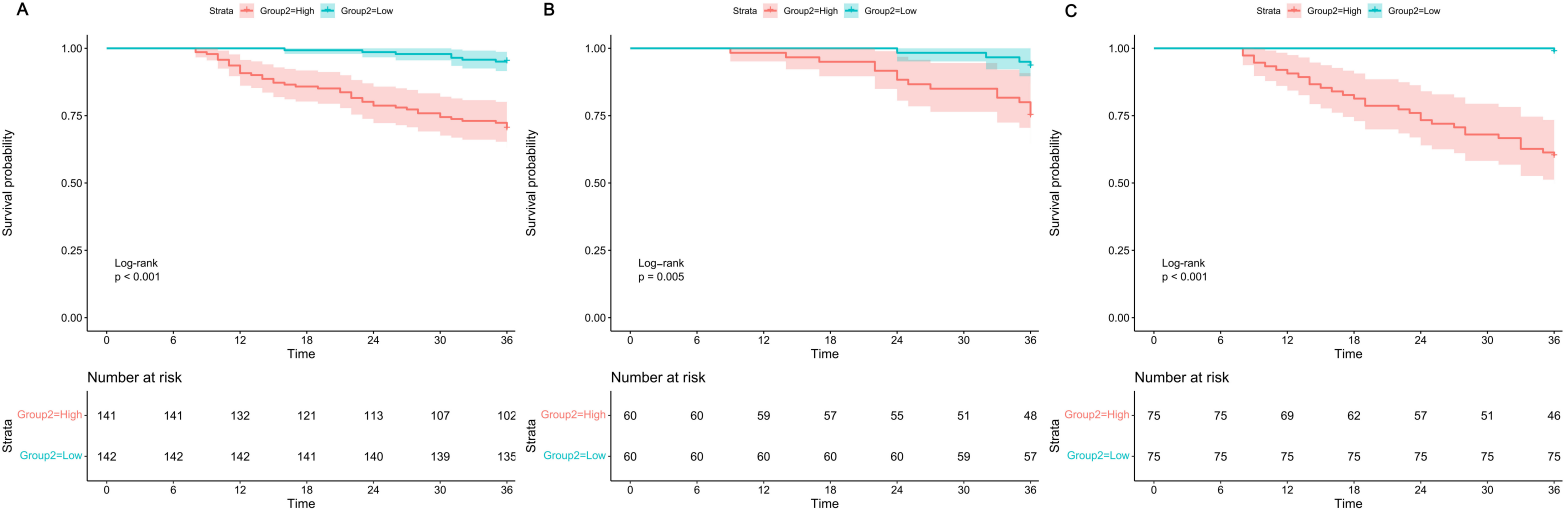
Kaplan-Meier curves stratified by model-predicted risk groups in the **(A)** PPMI training set, **(B)** PPMI internal validation set, and **(C)** local external validation cohort.

To further evaluate the model’s risk stratification ability, patients in each dataset were divided into high-risk and low-risk groups based on the median of the 36-month predicted first fall risk scores generated by the model. Kaplan-Meier curves were then used to compare the cumulative fall-free rates between the two groups. In the PPMI training set (Figure 8A), the 36-month fall-free survival probability was significantly lower in the high-risk group compared to the low-risk group (*P* < 0.001). In the PPMI internal validation set (Figure 8B), the model also effectively distinguished patients into different risk strata, with the fall-free survival curve for the high-risk group being significantly lower than that for the low-risk group (*P* = 0.005). In the local external validation cohort (Figure 8C), the model’s risk stratification capacity was further confirmed, as patients in the high-risk group had a significantly higher risk of experiencing a first fall than those in the low-risk group (*P* < 0.001). These results demonstrate that the fall-free survival probability was consistently and significantly lower in the high-risk group across the training, internal validation, and external validation sets.

## 4 Discussion

This study developed and externally validated a clinical prognostic model to predict the risk of a first fall within 3 years in fall-naive patients with early PD. The model integrates five readily accessible, independent predictors: lower BMI, presence of asymptomatic OH, lower MoCA score, GDS score > 5, and higher PIGD score. The model demonstrated good discrimination and generally acceptable calibration in the PPMI training set (optimism-corrected C-index 0.844) and internal validation set (C-index 0.768), and also showed good generalizability in an independent local Chinese cohort (C-index 0.825). This research provides clinicians with a novel, practical tool for the early identification of individuals at high risk for falls in early PD, to inform the implementation of targeted preventive strategies.

The pathophysiology of falls in PD is complex, involving dysfunction of cortical and subcortical motor and motor control networks, as well as disturbances in multiple neurotransmitter systems beyond dopamine (Fasano et al., 2017). The five risk predictors identified in our model are largely consistent with previously elucidated mechanisms underlying falls in PD, while also highlighting potential intervention targets. Firstly, lower BMI emerged as a risk factor for falls in our model. Several studies have indicated that low BMI can be a marker of frailty, sarcopenia, or malnutrition in PD patients, directly leading to reduced muscle strength, endurance, and balance, thereby increasing fall susceptibility (Bachmann and Trenkwalder, 2006; van der Marck et al., 2012). Secondly, the presence of asymptomatic OH highlights the importance of subclinical autonomic dysfunction in early PD fall risk. Routine screening of orthostatic blood pressure remains crucial, even if patients do not report typical symptoms like dizziness (Merola et al., 2016). Underlying cardiac sympathetic denervation and impaired cerebral autoregulation in asymptomatic OH may increase fall vulnerability during postural changes (Mei et al., 2024). Thirdly, a lower MoCA score, reflecting global cognitive decline, is a recognized key predictor of falls in PD (Allcock et al., 2009; Fasano et al., 2017). Specifically, cognitive impairments mediated by dysfunction of the basal forebrain and mesopontine cholinergic systems, particularly cortical cholinergic pathway disruptions affecting executive function, attention, and visuospatial abilities, can exacerbate difficulties in gait control under complex environments, hazard perception, and dual-task coordination, consequently increasing fall risk (Bohnen et al., 2009; Perez-Lloret and Barrantes, 2016; Wu et al., 2024). Crucially, a recent longitudinal clinicopathological study further confirmed that cognitive impairment and Alzheimer’s disease copathology, mainly amyloid-β deposition, act as robust risk factors for future falls and severe gait dysfunction in PD (Parmera et al., 2025). This finding reinforces the value of early cognitive assessment in risk stratification. Therefore, even if cognitive impairment in early PD primarily manifests as subjective cognitive decline or mild cognitive impairment, individuals with relatively lower neuropsychological test scores warrant careful attention regarding their fall risk. Fourthly, a GDS score > 5 indicates at least mild depressive mood. Depression, a common non-motor symptom in early PD, is associated with dysfunctional connectivity within fronto-striatal-limbic circuits (van der Marck et al., 2014). Depression can increase fall risk through various pathways, such as reduced motivation, decreased physical activity, fatigue, and diminished alertness (Ng et al., 2015), consistent with previous findings (Wood, 2002; Robinson et al., 2005; Bryant et al., 2012). Lastly, a higher PIGD score reflects relatively severe axial motor dysfunction present even in early disease and may herald a future transition towards the PIGD motor subtype. The associated increased fall risk is linked to cholinergic system degeneration related to microstructural changes in the pedunculopontine nucleus and weakened connections with the frontal cortex, as these structures and neural circuits are crucial for maintaining postural stability, balance control, and gait automaticity in daily activities (Craig et al., 2020). Notably, our previous research also found that even in early PD, patients with the PIGD subtype already display greater limitations in lower limb joint range of motion (Anis et al., 2025), which might be an early manifestation of their increased fall risk.

Our study provides a key distinction from previous fall prediction research by exclusively enrolling a fall-naive cohort of patients with early-stage Parkinson’s disease. This approach circumvents the circularity of using prior falls to predict a first fall, thereby establishing a model genuinely suited for primary prevention. Secondly, our model uses only demographic and clinical variables, obviating the need for expensive or invasive tests and avoiding biomarkers that are difficult to implement in routine clinical settings (especially primary care), which enhances its practical utility and potential for widespread clinical dissemination. Furthermore, considering that motor and non-motor complications such as FOG, dementia, and hallucinations are typically absent in early PD, the predictors identified by our model are based more on subtle, early-stage motor and non-motor manifestations. This focus on early, subtle features offers a critical window of opportunity for early intervention. The development and internal validation of our model were based on the large, multicenter, high-quality prospective PPMI cohort, and its external validation in an independent cohort strengthens its reliability and generalizability.

This model holds significant clinical implications for fall risk assessment and individualized management in patients with early PD. For high-risk patients, targeted preventive strategies can be proactively implemented based on the potentially modifiable risk factors identified in the model. Education for patients and their caregivers on fall risk and prevention should be prioritized (van der Marck et al., 2014). Individualized rehabilitation therapy, particularly gait and balance training, can help improve or compensate for early axial dysfunctions. Medication regimens should be reviewed and optimized to avoid or minimize drugs that may adversely affect cognitive load or contribute to blood pressure lability. For patients with low BMI, nutritional assessment and counseling, combined with appropriate resistance exercise, may improve physical function and muscle strength. For patients with depressive mood, effective depression management cannot only improve quality of life but also potentially reduce fall risk by enhancing motivation, activity levels, and alertness. For low-risk individuals, routine health education and continued follow-up can be provided, allowing for more rational allocation of healthcare resources.

This study also has some limitations. Although we performed external validation, the sample size of the external validation cohort was relatively small, which might affect the stability and statistical power of the validation results; consequently, further validation in larger, multicenter, and more diverse populations is warranted. Secondly, differences in baseline characteristics existed between the PPMI and local cohorts, with local cohort patients being older and having a longer disease duration. These differences might have contributed to the observed fluctuations in the model’s calibration during external validation and suggest that adapting or recalibrating the model may be necessary when applying it to populations substantially different from the development cohort. Notably, a substantial difference in treatment status existed between the cohorts, with the PPMI participants being predominantly drug-naive while the local cohort included patients already on dopaminergic therapy. On one hand, the model maintained robust performance in this medicated population, indicating that the selected predictors are stable risk factors largely independent of medication status. On the other hand, dopamine replacement therapy has a dual impact on motor control. Long-term non-physiological dopaminergic stimulation can eventually induce motor fluctuations and FOG, which are known factors that increase fall risk (Jansen et al., 2023). Therefore, the application and optimization of the model in medicated populations require specific attention. Additionally, the predictors included in this study relied primarily on clinical rating scales and patient reports. While this enhances clinical usability, these factors also introduce a degree of subjectivity and recall bias. Future research could explore the integration of easily accessible, non-invasive biomarkers, such as parameters from wearable devices or smartphone applications (Moreau et al., 2023), to further improve the model’s precision and objectivity. Finally, We acknowledge that the frequency of fall ascertainment varied between the two cohorts, with the local cohort assessed every 3 months compared to the 6-month interval in the PPMI cohort. This difference might introduce recall bias and potentially influence fall detection rates. However, given that the “first fall” is typically a salient event, we believe the impact on the model’s overall validity remains limited.

## 5 Conclusion

This study developed and validated a concise and practical clinical prediction model. Based on five readily accessible and potentially modifiable risk factors, this model can effectively aid clinicians in identifying fall-naive patients with early PD who are at risk of experiencing a first fall within the next 3 years. By identifying high-risk individuals at an early disease stage, this model provides a basis for implementing individualized preventive strategies, potentially delaying or preventing first falls, thereby mitigating their severe consequences and ultimately aiming to improve patients’ long-term prognosis and quality of life. In summary, this research offers a practical tool for risk stratification and individualized prevention of first falls in patients with early PD, with the potential to positively impact fall management in this vulnerable population.

## Data Availability

The data used in this study are available in the PPMI database (https://www.ppmi-info.org/). The data supporting this study’s findings are available from the corresponding author upon reasonable request.

## Footnotes

1
https://www.ppmi-info.org/

2
http://cran.r-project.org/
