# Supplementary material for: Development and validation of a clinical prediction model for first fall in early Parkinson’s disease: a study of two fall-naive cohorts
Source: Front Aging Neurosci. 2026 Feb 17;18:1735524. doi: 10.3389/fnagi.2026.1735524 (PMC12953559; doi:10.3389/fnagi.2026.1735524)
Supplement: Supplementary file 1 [file Data_Sheet_1.docx]

**Supplementary materials**

**Title** Development and validation of a clinical prediction model for first fall in early Parkinson's disease: A study of two fall-naive cohorts

**Author information**

Yu Wang*, Jianing Mei*, Yunzhe Tang*, Hongping Zhao, Zijun Wei, Qingliang Tao, Xueyi Han, Jiyuan Hu, Yunyun Zhang

*These authors contributed equally to this work and share first authorship.

**Affiliation** Department of Neurology, Yueyang Hospital of Integrated Traditional Chinese and Western Medicine, Shanghai University of Traditional Chinese Medicine, Shanghai, China

**Corresponding author**

Yunyun Zhang

Department of Neurology, Yueyang Hospital of Integrated Traditional Chinese and Western Medicine, Shanghai University of Traditional Chinese Medicine

Postal address: No. 110 Ganhe Road, Shanghai, China

Phone number: +86 18930569983

E-mail: [zhangyyshyy@shutcm.edu.cn](mailto:zhangyyshyy@shutcm.edu.cn)

**Contents**

[Supplementary Figure S1. Diagnostics for Multiple Imputation of Missing Data in the PPMI Cohort. 3](#_Toc23211)

[Supplementary Table S1. Baseline characteristics of the PPMI training and internal validation sets. 4](#_Toc7808)

[Supplementary Table S2. Complete results of univariable cox regression for in the PPMI training cohort. 6](#_Toc12287)

[Supplementary Table S3. Comparison of Model Performance between the initial model and the parsimonious model in the PPMI Cohort. 7](#_Toc28348)


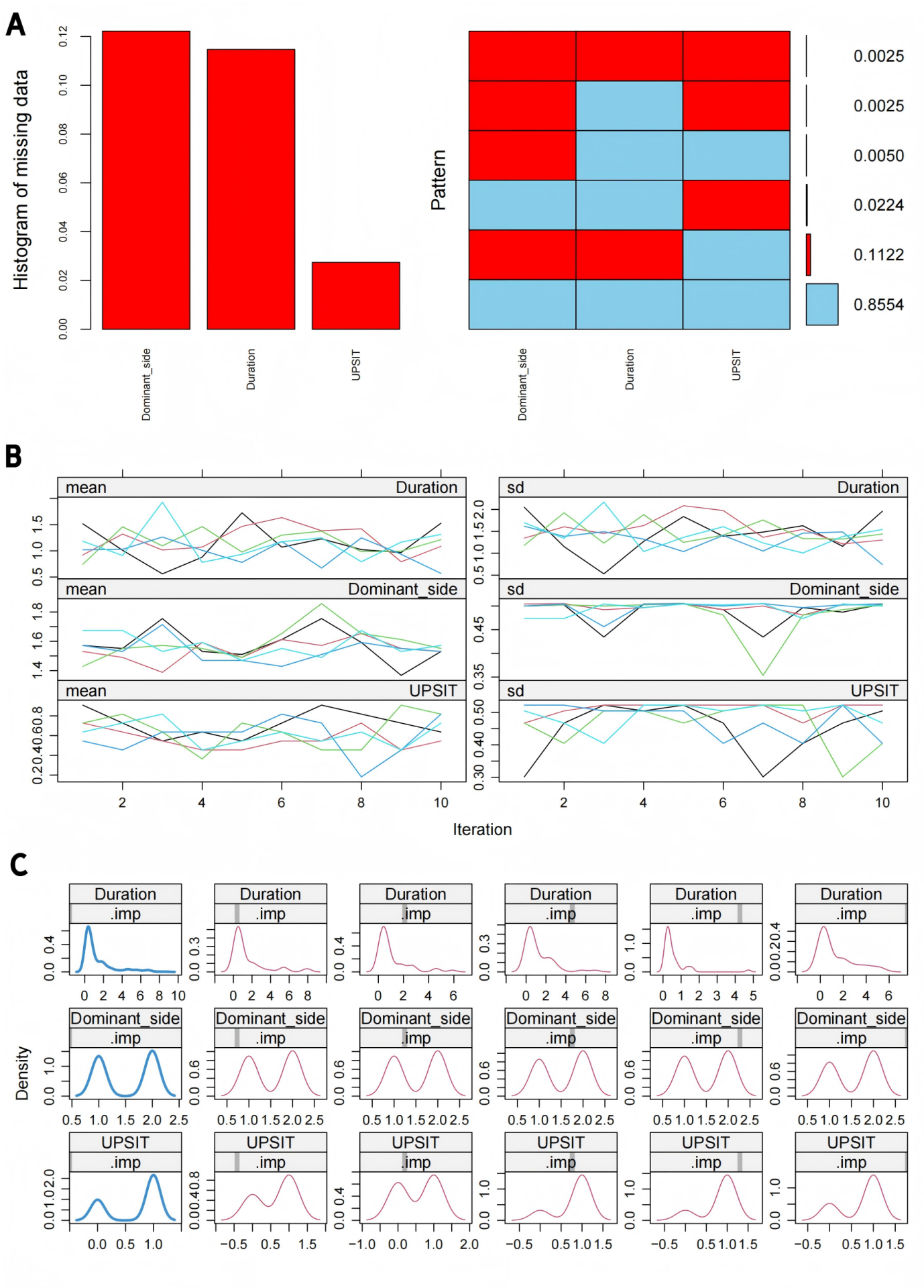


**Supplementary Figure S1.** Diagnostics for Multiple Imputation of Missing Data in the PPMI Cohort.

**Supplementary Table S1.** Baseline characteristics of the PPMI training and internal validation sets.

| **Variables** | **Overall (n=403)** | **Training set**  **(n=283)** | **Internal validation**  **set (n=120)** | **P value** |
| --- | --- | --- | --- | --- |
| **Demographics** | | | | |
| Age, years | 62.75 (55.89;68.70) | 62.12 (55.31;68.12) | 63.34 (57.38;69.73) | 0.053 |
| Sex, male, n(%) | 263 (65.26%) | 188 (66.43%) | 75 (62.50%) | 0.520 |
| Education, years | 16.00 (14.00;18.00) | 16.00 (14.00;18.00) | 16.00 (13.00;18.00) | 0.052 |
| BMI, kg/m² | 25.86 (24.09;28.92) | 25.78 (24.08;29.04) | 26.01 (24.16;28.64) | 0.785 |
| **Clinical characteristics** | | | | |
| Disease duration, years | 1.19 (0.57;2.17) | 1.21 (0.58;2.21) | 1.17 (0.56;1.88) | 0.373 |
| H&Y stage, n(%)  Stage 1  Stage 2 | 173 (42.93%)  230 (57.07%) | 120 (42.40%)  163 (57.60%) | 53 (44.17%)  67 (55.83%) | 0.828 |
| Family history, n (%) | 143 (35.48%) | 92 (32.51%) | 51 (42.50%) | 0.071 |
| Dominant side, right, n(%) | 215 (53.35%) | 156 (55.12%) | 59 (49.17%) | 0.324 |
| Motor subtype, n(%)  TD  Non-TD | 264 (65.51%)  139 (34.49%) | 184 (65.02%)  99 (34.98%) | 80 (66.67%)  40 (33.33%) | 0.838 |
| MDS-UPDRS Part Ⅰ | 5.00 (3.00;7.00) | 5.00 (3.00;8.00) | 4.00 (2.00;7.00) | 0.141 |
| Cognitive impairment, n(%) | 86 (21.34%) | 66 (23.32%) | 20 (16.67%) | 0.174 |
| Hallucinations and psychosis, n(%) | 11 (2.73%) | 9 (3.18%) | 2 (1.67%) | 0.518 |
| Depressed mood, n(%) | 93 (23.08%) | 71 (25.09%) | 22 (18.33%) | 0.179 |
| Anxious mood, n(%) | 140 (34.74%) | 103 (36.40%) | 37 (30.83%) | 0.338 |
| Apathy, n(%) | 63 (15.63%) | 44 (15.55%) | 19 (15.83%) | >0.999 |
| DDS, n(%) | 84 (20.84%) | 61 (21.55%) | 23 (19.17%) | 0.685 |
| Sleep problems, n(%) | 234 (58.06%) | 171 (60.42%) | 63 (52.50%) | 0.173 |
| Daytime sleepiness, n(%) | 219 (54.34%) | 156 (55.12%) | 63 (52.50%) | 0.708 |
| Pain, n(%) | 225 (55.83%) | 164 (57.95%) | 61 (50.83%) | 0.228 |
| Urinary problems, n(%) | 184 (45.66%) | 132 (46.64%) | 52 (43.33%) | 0.218 |
| Constipation problems, n(%) | 134 (33.25%) | 94 (33.22%) | 40 (33.33%) | >0.999 |
| Light headedness, n(%) | 104 (25.81%) | 74 (26.15%) | 30 (25.00%) | 0.907 |
| Fatigue, n(%) | 192 (47.64%) | 143 (50.53%) | 49 (40.83%) | 0.094 |
| MDS-UPDRS Part Ⅱ | 4.00 (2.00;7.00) | 4.00 (2.00;8.00) | 4.00 (2.00;6.00) | 0.230 |
| MDS-UPDRS Part Ⅲ | 18.00 (12.00;25.50) | 18.00 (12.00;25.00) | 17.00 (11.00;26.00) | 0.662 |
| Tremor score | 5.00 (2.00;7.00) | 4.00 (2.00;7.00) | 5.00 (1.75;7.00) | 0.674 |
| PIGD score | 1.00 (0.00;2.00) | 1.00 (0.00;2.00) | 1.00 (0.00;2.00) | 0.426 |
| Rigidity score | 3.00 (1.00;5.00) | 3.00 (1.00;5.00) | 2.00 (1.00;5.00) | 0.163 |
| Bradykinsia score | 8.00 (5.00;13.00) | 9.00 (5.00;13.00) | 7.00 (5.00;12.00) | 0.328 |
| SCOPA-AUT | 9.00 (5.50;12.50) | 9.00 (5.00;13.00) | 9.00 (6.00;12.00) | 0.890 |
| GDS>5, n(%) | 54 (13.40%) | 37 (13.07%) | 17 (14.17%) | 0.893 |
| STAI | 59.00 (48.00;74.00) | 60.00 (49.00;74.00) | 57.00 (47.00;74.00) | 0.177 |
| STAI State | 29.00 (23.00;38.00) | 29.00 (24.00;38.00) | 29.00 (23.00;37.25) | 0.485 |
| STAI Trait | 30.00 (24.00;37.00) | 30.00 (25.00;37.00) | 28.00 (23.00;36.00) | 0.073 |
| MoCA | 27.00 (26.00;29.00) | 27.00 (26.00;29.00) | 27.00 (26.00;29.00) | 0.173 |
| ESS | 5.00 (3.00;7.00) | 5.00 (3.00;7.00) | 5.00 (3.00;7.00) | 0.961 |
| RBDSQ | 3.00 (2.00;5.00) | 3.00 (2.00;6.00) | 3.00 (1.00;5.00) | 0.134 |
| Hyposmia, n(%) | 275 (68.24%) | 197 (69.61%) | 78 (65.00%) | 0.428 |
| Asymptomatic OH, n(%) | 58 (14.39%) | 41 (14.49%) | 17 (14.17%) | >0.999 |

Data are presented as median (Q1, Q3), or number (%).

**Abbreviations:** PPMI, the Parkinson’s Progression Markers Initiative; BMI, body mass index; H&Y stage, Hoehn and Yahr stage; TD, tremor dominant; MDS-UPDRS, the MDS-sponsored revision of the unified Parkinson’s disease rating scale; DDS, dopamine dysregulation syndrome; PIGD, postural instability and gait difficulty; SCOPA-AUT, scales for outcomes in Parkinson’s disease-autonomic dysfunction; GDS, geriatric depression scale; STAI, State-Trait anxiety index; MoCA, Montreal cognitive assessment; ESS, Epworth sleepiness scale; RBDSQ, REM sleep behavior disorder screening questionnaire; OH, orthostatic hypotension.

**Supplementary Table S2.** Complete results of univariable cox regression for in the PPMI training cohort.

| **Variables** | **Univariate cox regression** | |
| --- | --- | --- |
|  | HR (95% CI) | P value |
| Age | 0.984 (0.954-1.014) | 0.287 |
| Sex (male) | 0.776 (0.437-1.379) | 0.388 |
| Education | 0.949 (0.876-1.028) | 0.197 |
| BMI | 0.789 (0.709-0.878) | **<0.001** |
| Disease duration | 0.504 (0.351-0.724) | **<0.001** |
| H-Y stage (2) | 1.450 (0.805-2.610) | 0.216 |
| Family history | 1.316 (0.741-2.338) | 0.349 |
| Dominant side (right) | 1.212 (0.686-2.142) | 0.509 |
| Motor subtype (Non-TD) | 2.486 (1.416-4.365) | **0.002** |
| Cognitive impairment | 1.240 (0.657-2.337) | 0.507 |
| Hallucinations and psychosis | 4.158 (1.647-10.498) | **0.003** |
| Depressed mood | 0.753 (0.376-1.509) | 0.425 |
| Anxious mood | 0.840 (0.462-1.527) | 0.568 |
| Apathy | 1.498 (0.748-3.000) | 0.255 |
| DDS | 1.224 (0.638-2.348) | 0.542 |
| Sleep problems | 1.712 (0.921-3.182) | 0.089 |
| Daytime sleepiness | 1.267 (0.713-2.250) | 0.420 |
| Pain | 1.568 (0.863-2.849) | 0.140 |
| Urinary problems | 1.574 (0.894-2.773) | 0.116 |
| Constipation problems | 2.095 (1.196-3.668) | **0.010** |
| Light headedness | 1.716 (0.960-3.067) | 0.069 |
| Fatigue | 1.452 (0.821-2.567) | 0.200 |
| Tremor score | 1.012 (0.940-1.090) | 0.752 |
| PIGD score | 1.655 (1.437-1.906) | **<0.001** |
| Rigidity score | 1.136 (1.026-1.258) | **0.014** |
| Bradykinsia score | 1.039 (0.995-1.086) | 0.083 |
| SCOPA-AUT | 1.058 (1.020-1.097) | **0.003** |
| GDS (>5) | 4.773 (2.645-8.612) | **<0.001** |
| STAI State | 1.022 (0.996-1.049) | 0.101 |
| STAI Trait | 1.030 (1.002-1.058) | **0.037** |
| MoCA | 0.732 (0.658-0.815) | **<0.001** |
| ESS | 1.060 (0.985-1.142) | 0.119 |
| RBDSQ | 1.113 (1.028-1.204) | **0.008** |
| Hyposmia | 0.654 (0.368-1.163) | 0.148 |
| Asymptomatic OH | 7.769 (4.425-13.64) | **<0.001** |

**Abbreviations:** HR, hazard ratio; CI, confidence interval (95%); PPMI, the Parkinson’s Progression Markers Initiative; BMI, body mass index; H&Y stage, Hoehn and Yahr stage; TD, tremor dominant; DDS, dopamine dysregulation syndrome; PIGD, postural instability and gait difficulty; SCOPA-AUT, scales for outcomes in Parkinson’s disease-autonomic dysfunction; GDS, geriatric depression scale; STAI, State-Trait anxiety index; MoCA, Montreal cognitive assessment; ESS, Epworth sleepiness scale; RBDSQ, REM sleep behavior disorder screening questionnaire; OH, orthostatic hypotension.

**Supplementary Table S3.** Comparison of Model Performance between the initial model and the parsimonious model in the PPMI Cohort.

| **Performance metric** | **Model 1^a^** | **Model 2^b^** |
| --- | --- | --- |
| Training Set (n=283) | | |
| AIC | 448.78 | 455.78 |
| BIC | 470.66 | 474.01 |
| C-index (95% CI) | 0.859 (0.808-0.910) | 0.847 (0.788-0.906) |
| Internal Validation Set (n=120) | | |
| C-index (95% CI) | 0.763 (0.647-0.879) | 0.768 (0.656-0.880) |

**Abbreviations:** AIC, Akaike information criterion; BIC, Bayesian information criterion; CI, confidence interval; C-index, concordance index.

^a^ The Model 1 included BMI, PIGD, MoCA, GDS>5, asymptomatic OH, hallucinations and psychosis.

^b^ The Model 2 included BMI, PIGD, MoCA, GDS>5, asymptomatic OH.
